# Ultrasonographic measurement of gallbladder wall thickness in fasted dogs without signs of hepatobiliary disease

**DOI:** 10.1111/jvim.16810

**Published:** 2023-07-19

**Authors:** Carlos Martinez, Daniel Davies, Séamus Hoey, Robert E. Shiel, Emma J. O'Neill

**Affiliations:** ^1^ Department of Internal Medicine AÚNA Especialidades Veterinarias ‐ IVC Evidensia Valencia Spain; ^2^ Highcroft Referrals Bristol UK; ^3^ School of Veterinary Medicine University College Dublin, Belfield Co. Dublin Ireland; ^4^ School of Veterinary Medicine Murdoch University Perth Western Australia Australia

**Keywords:** canine, imaging, reference interval, ultrasonography

## Abstract

**Background:**

Ultrasound‐determined gallbladder wall thickness is widely used to aid in the diagnosis of gallbladder disease, but no reference values supported by published measurement data are available in dogs.

**Hypothesis/Objective:**

Establish normal thickness of the gallbladder wall in dogs.

**Animals:**

Fifty‐three dogs presented to a referral hospital and required abdominal ultrasound examination for reasons unrelated to primary hepatobiliary disease.

**Methods:**

Cross‐sectional observational study recruiting dogs requiring abdominal ultrasound examination. A standard sequence of gallbladder wall images was recorded for later review. Inclusion criteria were normal ultrasonographic hepatobiliary, pancreatic, and small intestinal findings. Exclusion was determined by 2 European College of Veterinary Internal Medicine (ECVIM)‐certified veterinary internists blinded to gallbladder wall thickness data. Dogs were excluded if they had inadequate medical records, a previous history of hepatobiliary, gastrointestinal, or pancreatic disease likely to impact the biliary system (eg, chronic vomiting, nausea, jaundice, diarrhea), unexplained increases in liver enzyme activities, hypoalbuminemia, or ascites. Gallbladder wall thickness was determined by 2 European College of Veterinary Diagnostic Imaging (ECVDI)‐certified veterinary radiologists working together to generate a consensus for each dog. The final output was the maximum normal wall thickness for this population of dogs.

**Results:**

The upper limit for gallbladder wall thickness in 53 fasted (8 hours) dogs <40 kg was 1.30 mm (90% confidence interval, 1.19‐1.41).

**Conclusions and Clinical Importance:**

Normal gallbladder wall thickness in dogs is lower than previously reported. Additional studies are required to determine potential effects of body weight and the optimal cut‐off to distinguish between healthy and diseased gallbladders.

**Abbreviations**
ECVDI: European College of Veterinary Diagnostic Imaging
ECVIM‐CA: European College of Veterinary Internal Medicine ‐Companion Animals
SD: standard deviation

## 1 INTRODUCTION

Ultrasonography is typically the initial diagnostic imaging method of choice for the evaluation of the biliary tract in dogs.^1^ In dogs presented with icterus, this assessment is aimed at establishing whether signs of biliary obstruction or biliary tract pathology are present, warranting further investigation.^2^ Having objective criteria by which to evaluate the biliary tract is crucial. Two commonly used measurements are gallbladder wall thickness and bile duct diameter, the former being used most often as an indicator of potential gallbladder disease.^3^

No clearly determined reference values have been established for the normal thickness of the gallbladder wall in dogs. The earliest, most widely cited article reported normal wall thickness as 2 to 3 mm.^4^ However, it provided no supporting data or references related to dogs. More recent publications have suggested that normal gallbladder wall thickness should be 1 to 2 mm, but again no supporting data were provided.^5^, ^6^

Most publications citing gallbladder wall thickness in dogs use the earlier values provided.^4^ However, discrepancy exists among this historically accepted value, clinical opinion,^5^ and indirect data in the literature, suggesting this value may be inappropriately high. First, two studies reported results from dogs without gallbladder disease. One evaluated biliary sludge in 42 dogs over 12 months and reported initial and final median gallbladder wall thicknesses of 1 mm (range, 0.44‐1.53 mm).^7^ The other was a study evaluating the safety of ultrasound‐guided cholecystocentesis^8^; mean wall thickness reported for the normal group (140 ultrasound evaluations) was 1.3 mm. The primary selection criterion for inclusion in this study was performance of cholecystocentesis and thus whereas the normal group consists of a biased population, it still suggests that the value of 3 mm is inappropriately high. Second, considering dogs with known gallbladder disease, many have gallbladder wall thickness <3 mm. For example, in 45 dogs with histologically confirmed gallbladder disease, only 23 had gallbladder wall thickening (>3 mm).^9^ Additionally, in 11 dogs undergoing cholecystectomy, none had increased gallbladder wall thickness on ultrasound examination (>3 mm) before surgery, despite histopathologically confirmed chronic cholecystitis in all 8 dogs sampled.^10^ Although some of these studies are several years old, using older ultrasound machines, transducers, and imaging software which may impact the accuracy and precision of measurements, it is evident that a lack of clarity remains on what constitutes an appropriate normal value for use in current practice.

Our aim was to objectively determine normal gallbladder wall thickness in dogs undergoing abdominal ultrasound examination for causes unrelated to primary hepatobiliary disease.

## 2 MATERIALS AND METHODS

### 2.1 Study design

Cross‐sectional observational study.

### 2.2 Case selection

A standard sequence of ultrasonographic gallbladder wall images was recorded prospectively under sedation using adult dogs presented to the veterinary diagnostic imaging service at University College Dublin Veterinary Hospital for causes unrelated to primary hepatobiliary disease over the study period (February 2018 to October 2019). Dogs were required to have normal ultrasonographic hepatobiliary, pancreatic, and small intestinal findings as well as no evidence of biliary obstruction, cholelithiasis, gallbladder mucocele, irregularities of the gallbladder wall, and no gallbladder content other than mobile echoic sediment consistent with biliary sludge. To minimize the possibility of pseudothickening of the gallbladder wall resulting from small gallbladder volume,^11^ all dogs were fasted for a minimum of 8 hours before ultrasonographic examination. The sedation protocol was not recorded as part of this study, but the standard protocol in our hospital is to administer 2 to 3 μg/kg medetomidine combined with 0.3 mg/kg butorphanol IV.

After imaging, the signalment, medical history (including medications), laboratory data, and ultrasonographic reports (blinded to wall thickness) were retrospectively reviewed. Dogs were excluded if they had inadequate medical records, a previous history of hepatobiliary, gastrointestinal, or pancreatic disease likely to impact the biliary system (chronic vomiting, nausea, jaundice, or diarrhea) or increased liver enzyme activities (>3‐fold the upper limit of the reference interval). To maximize the population available for evaluation and more accurately reflect the population that would typically be presented for this type of evaluation, dogs were retained if they had defined diseases affecting the gastrointestinal system thought unlikely to impact the gallbladder and biliary tract (eg, gastric ulceration, esophagitis) or those that had increased liver enzyme activities that could be explained by systemic diseases (eg, diabetes mellitus) or administration of drugs (eg, corticosteroids, phenobarbital). These cases were designated as complex cases in later analysis. Finally, dogs with inadequate image quality were excluded along with those with hypoalbuminemia (to exclude the possibility of gallbladder wall edema)^5^ or ascites to avoid potential impact on the accuracy of measurements.^5^

At the start of the data analysis phase, blinded to wall thickness findings, final cases were sub‐categorized as “simple” or “complex” by consensus of 2 European College of Veterinary Internal Medicine (ECVIM)‐certified internists (CM and EON) reflecting the presence or absence of intercurrent medical conditions that had the potential to influence gallbladder wall thickness. This categorization was based on presenting clinical signs (presence or absence of systemic signs such as fever or lethargy), clinicopathological findings (eg, liver enzyme activities >3‐fold the upper reference interval or normal), final diagnosis, and number of co‐morbidities present at the time of evaluation (see Table S1). Examples of cases sub‐categorized as complex included dogs diagnosed with renal failure, immune‐mediated hemolytic anemia, hypo‐ or hyperadrenocorticism, and multicentric lymphoma. This process permitted later evaluation of the influence, if any, of intercurrent disease on gallbladder wall thickness.

### 2.3 Imaging protocol

Ultrasound examination was performed using a LOGIQ E9 (General Electric) ultrasound machine, with 1 of 2 multifrequency transducers (ML6‐15 MHz linear transducer or C2‐9 MHz convex transducer). Transducer selection was dictated by dog size using the highest possible frequency to optimize image quality. The abdominal wall was clipped and acoustic coupling gel was applied to the skin. Imaging was performed in left lateral recumbency and the gallbladder was imaged via a right intercostal approach. Image depth was optimized by the ultrasonographer for the gallbladder wall. A single focal zone was centered at the level of the gallbladder wall nearest to the ultrasound transducer. Both static images and cine loops were acquired and exported to a workstation, in digital imaging and communications in medicine (DICOM) standard format for later measurement.

Images were reviewed by 2 European College of Veterinary Diagnostic Imaging‐certified radiologists (SH and DD) to generate a consensus value for thickness of the gallbladder wall most superficial to the transducer. Thickness was measured in millimeters to 2 decimal places using medical imaging software (Horos Imaging Software v3.3.5, Open‐Source License; Version 3 [LGPL‐3.0]) with measurements performed wherever possible using static images. When static images were deemed of insufficient quality, measurements were made from frozen cine loop frames. All data were recorded in a pre‐designed spreadsheet (Microsoft Excel) for later analysis.

### 2.4 Statistical analysis

Data were assessed for normality using the D'Agostino and Pearson test and evaluation of QQ plots. Data that did not deviate statistically from normal were described by mean (±SD) and non‐normally distributed data as median (range). Gallbladder wall measurements did not statistically deviate from a normal distribution, and outliers were identified by calculation of *z*‐scores. Gallbladder wall thicknesses with an associated *z*‐score > 2.5 were excluded. The upper limit for gallbladder wall thickness was estimated as the point below which 95% of values would be expected (mean + 1.645 × SD). The 90% confidence interval (CI) for this value was determined as described previously.^12^ A Student's *t*‐test was used to compare mean gallbladder wall thickness between the simple and complex groups, and any associations between gallbladder wall thickness and body weight, age, and sex were assessed using simple linear regression.

## 3 RESULTS

**FIGURE 1:**
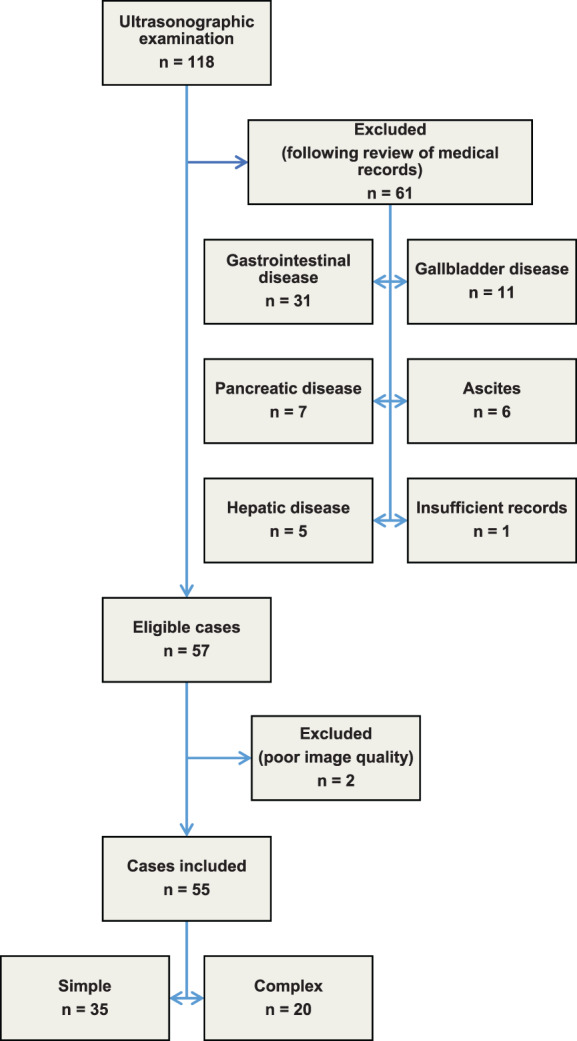
Flow chart showing recruitment and allocation of cases.

One‐hundred eighteen dogs undergoing ultrasonographic examination for various reasons initially were identified. Sixty‐one dogs were excluded after review of the medical records and, of the remaining 57 dogs, 2 additional cases were excluded for inadequate recorded image quality (Figure 1).

**FIGURE 2:**
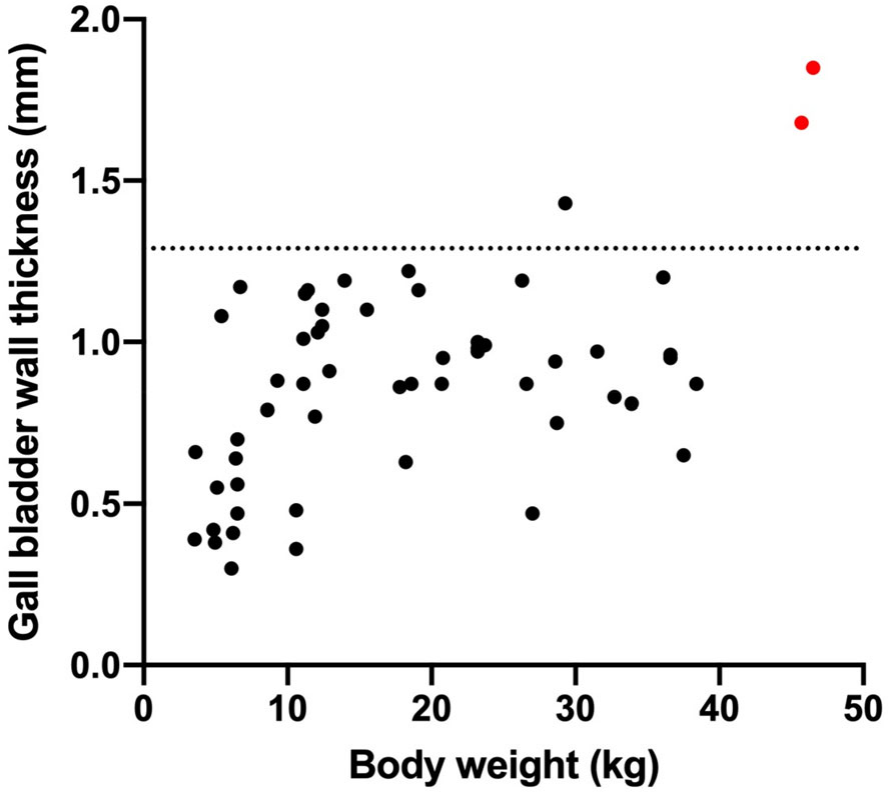
Gallbladder wall thickness in 55 dogs presented for abdominal ultrasound examination for causes unrelated to primary hepatobiliary disease. Results for individual dogs are shown as dots with those in red excluded due to high *z*‐scores (>2.5). The dotted line shows the value for gallbladder wall thickness estimated as the point below which 95% of values would be expected (mean + 1.645 × SD).

Gallbladder wall measurements were available for 55 dogs (Figure 2). Values did not significantly deviate from a normal distribution (*P* = .16). Two outlying gallbladder wall values were identified with *z*‐scores of 3.05 and 2.51. These values were identified in 2 dogs weighing >45 kg; all other animals weighed <40 kg (median, 13.95 kg; range, 3.55‐38.4 kg). After exclusion of these values, mean gallbladder wall thickness was 0.85 ± 0.27 mm. Twenty‐eight dogs were male (19 castrated) and 25 female (19 spayed). Ages ranged from 0.6 to 14.4 years (mean ± SD, 6.9 ± 3.75 years). Dogs consisted of the following breeds: Yorkshire terrier (n = 5); Jack Russell (n = 4); cocker spaniel (n = 4); bichon frise (n = 4); German shepherd (n = 3); Labrador (n = 3); beagle (n = 2); boxer (n = 2); golden retriever (n = 2); lurcher (n = 2); Maltese (n = 2); miniature schnauzer (n = 2); cross breed (n = 8); and 1 each of 10 other breeds.

**FIGURE 3:**
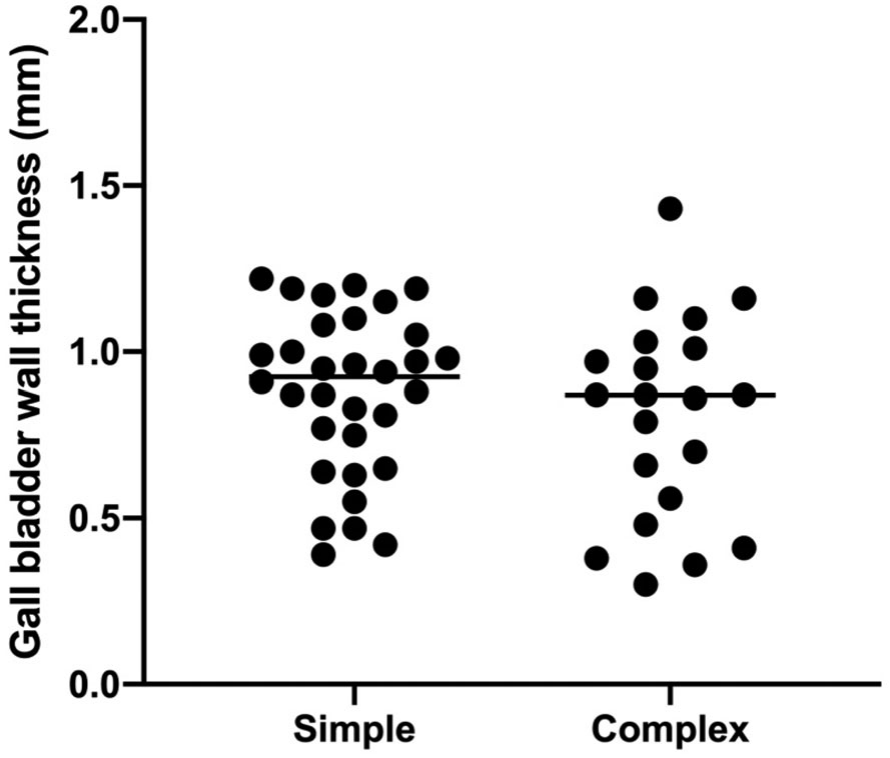
Gallbladder wall thickness in 53 dogs presented for abdominal ultrasound examination for causes unrelated to primary hepatobiliary disease with cases classified as simple or complex based on clinical presentation. Results for individual dogs are shown as dots with dogs classified into either the simple or complex groups. This classification was performed before data analysis, blinded to the gallbladder wall thickness. Details of the reasons for each case classification are available in Table S1. The solid black lines show mean gallbladder wall thickness for each group. No significant difference was found between the means for each group (*P* = .36).

The upper limit for normal gallbladder wall thickness was 1.30 mm (90% CI, 1.19‐1.41). Body weight was a significant but very weak predictor of gallbladder wall thickness (*R* ^2^ = .138; *P* = .01). Neither sex nor age predicted gallbladder wall thickness (*P* = .17 and .65, respectively). Mean gallbladder wall thickness for the simple and complex cases was not significantly different (*P* = .36; Figure 3).

## 4 DISCUSSION

We report an upper value for normal gallbladder wall thickness in fasted dogs <40 kg of 1.30 mm (90% CI, 1.19‐1.41). To date, gallbladder wall thickness typically has been evaluated against an anecdotally reported measurement, without a formal evidence base.^4^ Gallbladder wall thickness identified in our study is considerably less than the widely cited 3 mm value,^4^ and more aligned with recent indirect evidence presented above^7^, ^8^, ^10^ and the statement that based on clinical experience, most normal gallbladder walls in dogs and cats are <1 mm.^5^ Ultrasound evaluation of the biliary tract is an important component of the clinical assessment of dogs presented with jaundice^13^ or other signs of liver disease,^14^ and the use of a lower reference value for gallbladder wall thickness may prompt further investigations in dogs with measurements that previously would have been considered within the reference interval.^13^

The normal gallbladder wall appears as a thin echogenic line during ultrasound examination, and may be difficult to identify in its entirety when not diseased.^5^, ^7^ Gallbladder wall thickening can occur either diffusely or focally, and may reflect gallbladder disease, systemic illness, iatrogenic causes, or be artifactual in nature.^5^, ^15^, ^16^ Artifactual increases in wall thickness primarily relate to imaging technique or increases that can occur when free fluid surrounding the gallbladder mimics diffuse wall thickening.^4^, ^5^ The type of transducer used, orientation of the sound beam relative to the gallbladder wall, and the extent of gallbladder distension all impact wall thickness.^5^ Consequently, animals were fasted (> 8 hours) to ensure adequate gallbladder volume, and a standardized approach was used and is recommended to allow direct comparison across dogs and to facilitate application of our obtained value in clinical practice. Another important consideration is the sedation protocol used before imaging. Because dexmedetomidine has been documented to result in significant increases in gallbladder wall thickening in normal dogs,^16^ alpha‐2 agonists should be avoided or used judiciously, avoiding high doses, before gallbladder measurement, as was the case in our study.

Several mechanisms are thought to contribute alone or in combination with each other to actual increases in gallbladder wall thickness. Examples include changes in tissue composition, such as accumulation of inflammatory infiltrates, or edema relating to changes in the balance between intravascular oncotic pressure and portal hydrostatic pressure, changes in vascular permeability, and alterations in lymphatic drainage.^5^ The relative combination of each likely reflects both the disease process and its chronicity. Focal wall thickening typically reflects primary gallbladder pathology, such as benign or malignant neoplasia^11^ or cystic hyperplasia.^17^ Diffuse wall thickening has been associated with several other differential diagnoses reflecting the pathophysiology described above. Systemic conditions reported to be associated with diffuse gallbladder wall thickening in dogs include sepsis,^18^ right‐sided heart failure,^19^, ^20^ anaphylaxis,^21^ and immune‐mediated hemolytic anemia.^22^ Hypoalbuminemia also has been reported to be associated with gallbladder wall thickening,^19^ although more recently this association has been questioned.^23^ These reports informed the exclusion criteria applied in our study and are important to consider when using gallbladder wall thickness to aid clinical decision making.

Two outliers were identified in our study. Outliers can occur for several reasons, broadly classified as data entry or measurement errors, sampling issues, or natural variation. In our study, the 2 animals with outlying gallbladder wall thicknesses were also the 2 heaviest animals. A decision was made to exclude these values and limit the study to animals <40 kg, avoiding potentially inaccurate assumptions based upon inclusion of a very small number of heavier dogs. Notably, these dogs fell into the simple grouping. Additional studies are warranted to evaluate gallbladder wall thickness in larger dogs.

Our study had several limitations. First, healthy dogs were not used to derive gallbladder wall thickness. A group of dogs presented for abdominal ultrasound examination unrelated to hepatobiliary disease with final diagnoses including a wide range of illnesses was selected for this purpose. Although this approach could be seen as a limitation, we considered it to be more representative of the population that would typically be evaluated, avoiding selection bias that would have resulted from choosing entirely healthy animals. The pregrouping of animals into simple and complex groups before analysis and blinded to gallbladder wall thickness allowed retrospective confirmation that broadening inclusion criteria did not impact wall thickness because no significant difference was found between these 2 groups. Second, the possibility of intra‐ and inter‐observer measurement error during evaluation of the gallbladder walls cannot be ignored, particularly when assessing such small measurements. To minimize the possible influence of these effects, all measurements reflect values agreed between 2 European College of Veterinary Diagnostic Imaging (ECVDI)‐certified radiologists. Third, because gallbladder wall histopathology was not performed on any animal, the potential for occult gallbladder disease existed in some animals. Finally, inclusion of a larger number of dogs, with a broader range of body weights, would have allowed more accurate determination of an optimal reference limit, and determination if different values should be used depending upon the body weight of an individual dog.

These results have important clinical implications. First, they provide a valuable benchmark against which to evaluate increases in gallbladder wall thickness. The availability of an objective measure to apply in this setting should remove current uncertainty and the tendency to use different reference values. Because these results identify a much lower normal value than typically applied (3 mm), they are likely to have a marked impact on the sensitivity of gallbladder ultrasound examination for the diagnosis of gallbladder diseases, and in particular cholecystitis. Although indirect circumstantial evidence supports this suggestion, such as the far higher proportion of cases with histopathologically confirmed cholecystitis than was identified ultrasonographically in a series of cholangitis cases^24^ or a study with similar findings,^10^ further work is required to investigate the sensitivity and specificity of this measurement for the identification of cholecystitis in dogs.

In conclusion, we scientifically determined a value for normal gallbladder wall thickness in dogs. Our results showed that in fasted dogs <40 kg, normal gallbladder wall thickness should be ≤1.30 mm. This value is much lower than the previously used value and thus has important clinical implications.

## CONFLICT OF INTEREST DECLARATION

Authors declare no conflict of interest.

## OFF‐LABEL ANTIMICROBIAL DECLARATION

Authors declare no off‐label use of antimicrobials.

## INSTITUTIONAL ANIMAL CARE AND USE COMMITTEE (IACUC) OR OTHER APPROVAL DECLARATION

Study exempted from full ethical review by the Animal Research Ethics Committee (AREC), University College Dublin, AREC‐18‐21‐ONeill.

## HUMAN ETHICS APPROVAL DECLARATION

Authors declare human ethics approval was not needed for this study.

## Supporting information


Table S1:
