# Supplementary material for: Ultrasonographic measurement of gallbladder wall thickness in fasted dogs without signs of hepatobiliary disease
Source: J Vet Intern Med. 2023 Jul 19;37(5):1766–71. doi: 10.1111/jvim.16810 (PMC10473047; doi:10.1111/jvim.16810)
Supplement: Supplementary file 1 — Table S1: [file JVIM-37-1766-s001.pdf]

Supplementary information

Breed	Final diagnosis	Reason for allocation to simple / complex
Jack Russell terrier	Hypothyroidism	Normal bloodwork<br>No U/S changes
Bichon frise	Urinary bladder neoplasia	Increased ALP activity<br>Heterogeneous liver parenchyma with multiple hyperechoic nodules
Irish Glen of Imaal terrier	Anal sac adenocarcinoma	Increased ALP activity<br>hepatomegaly, heterogeneous parenchyma with a hypoechoic nodule
Golden retriever	Mast cell tumour (left stifle) with splenic involvement	Increased ALP activity
Maltese terrier	Pelvic fractures	Normal bloodwork<br>No liver U/S changes
Jack Russell terrier	Immune-mediated thrombocytopenia	Normal bloodwork<br>No liver U/S changes
Yorkshire terrier	Atopic dermatitis	Normal bloodwork<br>No liver U/S changes
Bichon Frise	Hyperadrenocorticism	Acute onset vomiting<br>Increased ALP and ALT activities
Cavalier King Charles Spaniel	Chiari-like malformation	Normal bloodwork<br>No liver U/S changes
Bichon frise	Suspected haemolytic process	Increased ALP and ALT activities
German shepherd	Hypoadrenocorticism	Normal bloodwork<br>No liver U/S changes
French bulldog	Multicentric lymphoma	Increased ALP and ALT activities
Labrador retriever	Renal failure	Mild increase ALP activity
Cross breed	Splenic extramedullary haematopoiesis	Fever<br>Increased ALP and ALT activities<br>Heterogeneous liver parenchyma
Miniature schnauzer	Gastric ulceration	Gastric U/S changes
Maltese terrier	Perineal hernia	Normal bloodwork<br>No liver U/S changes
Cross breed	Anal sac adenocarcinoma	Normal bloodwork<br>No liver U/S changes
Golden retriever	Hyperadrenocorticism	Normal bloodwork<br>No liver U/S changes
Jack Russell terrier	Splenic histiocytic sarcoma	Increased ALP and ALT activities<br>Multifocal hepatic mineralisation
German shepherd	Inflammatory spinal disease	Normal bloodwork<br>No liver U/S changes

Weimaraner	Immune-mediated polyarthritis	Normal bloodwork
Cross breed	Urothelial carcinoma	Increased ALP activity<br>Diffuse liver nodules
Cocker spaniel	Immune-mediated haemolytic anaemia	Normal bloodwork<br>No liver U/S changes
Cross breed	Multicentric lymphoma	Normal bloodwork<br>No liver U/S changes
Cross breed	Urinary tract infection	Normal bloodwork<br>No liver U/S changes
German shepherd	Immune-mediated haemolytic anaemia	Normal bloodwork<br>No liver U/S changes
Bichon frise	Suspected thoracolumbar/lumbosacral disease	Normal bloodwork<br>No liver U/S changes
Rottweiler	Necro-suppurative myositis	Increased ALP activity
Springer spaniel	Evan´s syndrome	Normal bloodwork<br>No liver U/S changes
Beagle	Cutaneous extramedullary plasmacytoma	Normal bloodwork<br>No liver U/S changes
Yorkshire terrier	Oesophagitis secondary to ingestion of foreign body	Acute vomiting and regurgitation
Alaskan malamute	Acute kidney injury	Normal bloodwork<br>No liver U/S changes
Boxer	Intra-axial brain neoplasia	Normal bloodwork<br>No liver U/S changes
Labrador retriever	Limb osteosarcoma	Normal bloodwork<br>No liver U/S changes
Boxer	Cutaneous mast cell tumours	Normal bloodwork<br>No liver U/S changes
Pug	Diabetes mellitus	Increased ALP activity
Cross breed	Lymphoplasmacytic colitis and proctitis	No liver U/S changes
Yorkshire terrier	Diabetes mellitus and hyperadrenocorticism	No liver U/S changes
Labrador retriever	Diabetes mellitus	Increased ALP and ALT activities<br>Diffuse hepatic nodules
Cocker spaniel	Mammary carcinoma	Normal bloodwork<br>No liver U/S changes
Lurcher	Urothelial carcinoma	Normal bloodwork<br>No liver U/S changes
Irish red setter	Suspected urethral sphincter mechanism incompetence	Normal bloodwork<br>No liver U/S changes
Cocker spaniel	Multicentric lymphoma	Increased ALP activity<br>Diffuse hepatic nodules
Cocker spaniel	Nephrotoxicity	Normal bloodwork<br>No liver U/S changes

Yorkshire terrier	Cranial cruciate ligament rupture	Increased ALP activity
Yorkshire terrier	Suspected primary polydipsia / atypical hyperadrenocorticism	Increased ALP activity<br>Diffuse hepatic nodules
Jack Russell terrier	Urothelial carcinoma	Increased ALP activity<br>Diffuse hepatic nodules
Shih tzu	Splenic histiocytic sarcoma	Normal bloodwork<br>No liver U/S changes
Boxer	Urinary tract infection	Normal bloodwork<br>No liver U/S changes
Beagle	Glomerular disease	Increased ALP activity
Miniature schnauzer	Suspected vasculitis	Increased ALP activity
Staffordshire bull terrier	Mast cell tumour (right metacarpus)	Normal bloodwork
Cross breed	Immune-mediated haemolytic anaemia	Increased ALP and ALT activities
Cockapoo	Suspected urethral sphincter mechanism incompetence	Normal bloodwork<br>No liver U/S changes
Lurcher	Suspected urethral sphincter mechanism incompetence	Normal bloodwork<br>No liver U/S changes

Prior to data analysis, dogs were classified as either ‘simple’ or ‘complex’ by ECVIM-certified internists blinded to the gallbladder wall thickness. This table provides a summary of the final diagnosis and the reason for the classification of each case

Complex cases are highlighted in grey. U/S ultrasound
